# Perspective on the challenges and opportunities of accelerating drug discovery with artificial intelligence

**DOI:** 10.3389/fbinf.2023.1121591

**Published:** 2023-02-23

**Authors:** John P. Santa Maria, Yuan Wang, Luiz Miguel Camargo

**Affiliations:** ^1^ Data and Translational Sciences, UCB Biosciences Inc., Cambridge, MA, United States; ^2^ 17-09, LLC, Wellesley, MA, United States

**Keywords:** artificial intelligence, machine learning, drug discovery and development, data, compute

## 1 Introduction

Technology has long been a driver of innovation and improvement in drug discovery (Gershell and Atkins, 2003; Pina, et al., 2009). Advancements within fields such as chemical engineering, high-throughput experimentation, and molecular biology have transformed the process of drug hunting from serendipitous discovery within historic dye collections to methodical science with empowered understanding of medicines’ impacts on human health. Despite improvements in scope and cost-efficiency brought on by adopted technologies, our industry follows “Eroom’s Law”, an observed exponential decay in FDA new drug approvals per billion dollars of R&D investment (Scannell, et al., 2012), highlighting a key expectation-reward gap between technological investments and payout in therapeutics.

Drug discovery is, in practice, a chain of challenging decisions across diverse disciplines. In considering the journey of a therapeutic candidate from administration to *in vivo* target engagement, a discovery team must address numerous biological and chemical questions. No single technology or dataset has emerged that is powerful enough to tackle all of these questions in aggregate, and we must often drive decisions using surrogate or partial readouts in the absence of ethical and/or practical measurements (Scannell and Bosley, 2016).

Artificial Intelligence (AI), a technology that has gained a lot of publicity and investment, is unique in its promise to impact multiple challenges across the drug discovery pipeline (Lounkine, et al., 2012; Vamathevan, et al., 2019; Bender and Cortés-Ciriano, 2021; Gupta et al., 2021; Paul, et al., 2021; Renaud and Wang, 2021; Kumar et al., 2023). On one hand, when appropriately applied, AI can help us leverage advances in laboratory techniques, generated data, and computational algorithms to make the best decisions we can using the often incomplete information that we have. On the other hand, AI has already had some controversial and expensive failures in the industry, such as Watson AI for automated disease diagnosis and the clinical trial failure of Exscientia’s DSP-1181, touted as the first AI-designed drug (O’Leary, 2022; Raleigh, 2022). History teaches us that technology integration happens in the context of the present-day and thus implementation is neither seamless nor immediate. Knowing the strengths and weaknesses of AI can help ensure its correct application and reduce the risks of both over- and underinvestment.

## 2 Machine learning (ML) and AI in drug discovery

The recent procurement of diverse biological and chemical data generated by ‘omics technologies has fueled an AI revolution for biomedical sciences (Biswas and Chakrabarti, 2020). The charge for data-hungry ML is to help us extract value from these experiments: identifying patterns beyond human recognition, distilling large and/or complex datasets, and generating predictions to inform future experiments. The hope is that resulting ML models built on biochemical data capture underlying principles describing molecules and the behaviors of living systems with implications for disease amelioration. Some of these principles have direct translation to the drug discovery process, such as enabling the engineering of antibodies with improved target affinity and reduced immunogenicity (Bachas, et al., 2022). Others have less immediate applicability—for example, functional understanding of AI-predicted RNA spliceoforms (Jaganathan, et al., 2019) toward new targets may be limited by our understanding of the nuanced contexts of disease, and ML-informed protein structures (Jumper, et al., 2021; Lin, et al., 2022) may not guarantee the identification of therapeutic binders, due to limitations such as the synthesizable chemical space of today’s screening libraries. Nevertheless, each advance improves our ability to answer key scientific questions and make informed decisions, with industry impact proportional to the target and disease focus areas in which it can be applied.

The ability of AI to address diverse problems and data types is enabled in part by the modularity of ML architectures. In practice, observed success of an AI method for one task may lead to rapid trialing, tailoring, or even direct transfer of encoded modules and learnings for the next. However, domain-specific performance boosts are often achieved using customized scoring functions or connectivities and processing steps adapted to the input data. For example, advanced algorithms like AlphaFold2 and ESMFold illustrate how inputting amino acid sequence alignments together with procedures for facilitating information flow between modules and continuous refinement significantly improved prediction of protein structure (Jumper, et al., 2021; Lin, et al., 2022). Understanding input requirements of data and algorithms can help ensure correct application of these kinds of ML approaches to impact drug discovery.

## 3 Requirements and challenges for using AI

**FIGURE 1:**
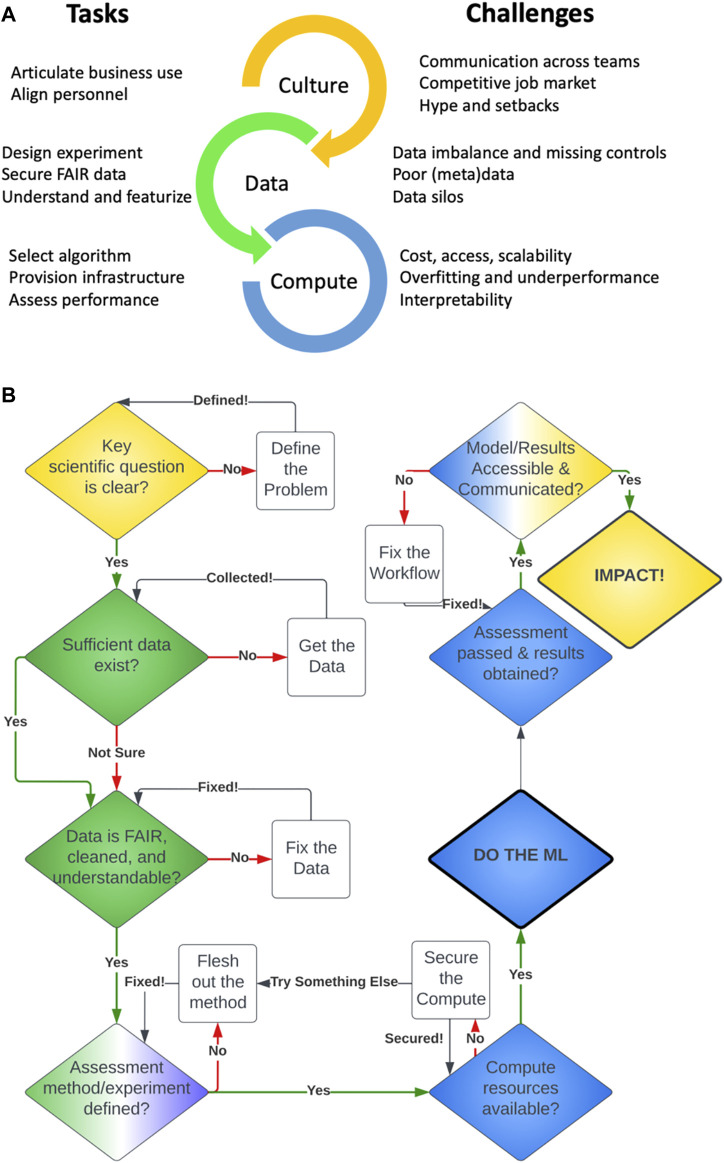
**(A)** Tasks required and challenges encountered in ML applications for drug discovery. **(B)** A decision flowchart for applying ML in drug discovery.

The existence of challenges in drug discovery does not guarantee AI as an immediate and practical solution. Required ingredients of data, compute, expertise, business utility, and a digital-savvy culture must first be assembled with conscious investment to first ensure readiness and implementability (Figure 1). Deficiencies in any of these elements limit the value we can generate with AI.

As the input for ML algorithms, the shape, quality, scope, and quantity of data matter. Data can be organized in many ways—for example, time series or multidimensional measurements can be stored within one wide or multiple narrow arrays—and conscious data shaping can mitigate misinterpretation and downstream reorganization. Meanwhile, quantity and type of input data can dictate AI method selection. Data such as protein structures or microscopy images often describe multi-dimensional interactions concentrated within localized contexts, and architectures best suited to learn from these data are often different from those that best learn from categorical or single point measurements. Augmenting limited data with new datasets or experiments can increase ML power and scope, though caution is required as diverse sources may introduce unique biases and noise. Alternatively, algorithms can work to mitigate deficiencies in data, such as inferring missing values, or over/under-sampling to improve balance in learning (Chawla, et al., 2002; Hessler and Baringhaus, 2021; Irwin, et al., 2021). Spending time understanding the data can be helpful in assessing when a problem is ready to be tackled with AI or when investment is needed in collecting data that would better inform the task at hand. Failure to assess and address data can decrease the accuracy, confidence, reproducibility, and applicability of downstream ML applications (United States Government Accountability Office, 2019), sometimes propagating even beyond to patient outcomes (Seyyed-Kalantari, et al., 2021).

Regardless of the format of input data, featurization, or the description of entities/samples based on measured or inherent properties, must be carefully performed to best complement the intended ML application. For example, small molecules can be featurized in many ways (David, et al., 2020): using SMILES, text-based descriptors of 2D structures; using atom coordinates describing 3D conformation; using calculated or measured physicochemical descriptors such as LogD and pKa; or even using methods such as extended-connectivity fingerprints (Rogers and Hahn, 2010) that iteratively capture atom connectivities. Choice of featurization affects result interpretability and actionability—ML to inform QSAR decisions for medicinal chemists might favor physicochemical descriptors that highlight relevant property changes for synthetic modifications, while ML for high-throughput screeners might benefit from topological descriptors to learn diverse scaffold hits within an experiment. Evaluating feature similarity is particularly important for generative chemistry endeavors, where AI-proposed molecules should be compared with training data to evaluate novelty (Walters and Murcko, 2020; Wills, 2021).

One often under-addressed component of data quality is metadata. Because biological systems are complex, drug discovery experiments often possess conditional features whose importance is realized only after subsequent measurements. Capturing information such as a cell line, associated genetic engineering, and time of measurements can enable downstream AI-based detection of systematic measurement biases, such as batch effects (Sprang et al., 2022), and meta-analyses across experiments. Metadata can also include interpretations of data—what were the hits in an experiment and what criteria were employed in their selection? This information is historically under-reported and difficult to assemble retrospectively. As a core enabler of findable, accessible, interoperable, and reusable (FAIR) data tenets (Wilkinson, et al., 2016), metadata should also include source information, especially when integrating external data. Because drug discovery data are diverse and acquired across disciplines and sources, (meta) data are rarely uniform. But, extra investment in data assembly and an infrastructure to enable data storage and sharing is often worth it, as failure to capture both data and metadata in the present may impair utility of data assets in future AI applications.

Compute, comprising algorithms and infrastructure, is the second requirement for AI. Compute facilitates both the execution of AI and interpretation of AI outputs. Computational algorithms should be selected with careful consideration of the data available and the problem at hand. Metrics, such as accuracy and area under the receiver-operator curve (AUROC) can facilitate quantitative evaluation of algorithm performance and comparison of new methods to the state of the art. Calculating these metrics requires definition of ground truth, which is supported by upfront investment in experimental design and inclusion of labeled control sample inputs. Published benchmarking datasets and tasks, such as GuacaMol (Brown, et al., 2019) and MoleculeNet (Wu, et al., 2018) for small molecules, and TAPE (Rao, et al., 2019) for protein structures, help standardize and contextualize these assessments, but may not exist for specialized applications.

Some ML methods, especially deep learning, are data and compute intensive, which may limit their implementability within organizations. Nevertheless, sharing centralized computational and data infrastructure across groups can help reduce cost. Computing environments must also be secure to protect proprietary data and sensitive patient data. Dedicated support from IT experts and/or cloud platform CROs can better ensure the timely and secure assembly of data, algorithm, and computational infrastructure on demand; otherwise time spent on this is time scientists spend away from solving the problems at hand. This is a significantly underreported and critical issue, as poor support delays insight delivery, and can decouple ML with expected time frames of decision making in drug discovery projects.

As a final key ingredient, organizational culture defines how AI can be successfully deployed, informing its business use and intended application. In most organizations, the generation of required input data, execution of AI, and validation of its impact can fall within separate groups. This requires machine learners to establish functional relationships with these key stakeholders to understand business needs and engineer AI solutions. Well-maintained relationships ensure continuous vision of actionability and mitigate both the risks of misapplication of AI and mis-/over-interpretation of data and results.

As data consumers, AI/data scientists are often the translators that convert data generated by laboratory scientists into viable inputs for machine learning models. In return, data scientists must ensure models and their results are continuously accessible and shared back with benchtop scientists. The two groups must work together to establish standards and practices for data/model capture and utilization, ensuring today’s work informs smarter experiments tomorrow. AI-assisted Design-Make-Test and iterative screening cycles are great illustrations of how regular communication of data and ML results between groups or functions can accelerate hit identification and optimization (Pant, et al., 2018). Data and model siloing, especially in large organizations, impairs this communication and reduces accessibility and utility.

ML models are only useful if their outputs are actionable—in general, that they inform experiments or decision making toward the business impacts of developing a drug. As an example, for AI-generated compound hypotheses this can mean ensuring molecules are synthetically feasible and thus able to be tested. Business understanding also informs performance and time requirements for AI—a model must have higher specificity for hit identification if only a small number of generated compounds can be tested versus a large library. And, training a model for a week is infeasible if outputs are needed on shorter timescales. A breakdown in business understanding can lead to the problems of data scientists building hammers with no nails, or for securing screws.

Establishing a data-aware and AI-supportive culture can sometimes be a roadblock for AI utilization. This can manifest as organizational inertia or politics when AI automation obviates work that was previously performed with an established way of working, or when employees are asked to allot already limited time to new data initiatives. Hype and endorsements from executive leadership can be helpful in motivating diverse teams to support new AI endeavors, but can also lead to an expectation-reward error, as incidences of overpromising and under-delivering sever trust between computational and bench scientists.

To summarize, without good data, compute, people, and culture, including business utility, AI is not possible. Nor is AI the panacea for the absence of one of these components. Tapping into the true and sustainable impact of AI means maintaining these dependencies while tackling challenges that can arise.

## 4 Discussion—The future of AI in drug discovery

The generation of new quantities and types of biomedical data, together with continuous improvements in computation and lessons learned from ML applications, have driven evolution of the pharma AI landscape. There are key challenges ahead such as bringing ML to new data types and domains and in integrating diverse data to better inform current implementations. Some initial barriers to entry have fallen: democratization of data and algorithms with databases and code repositories have enabled sharing both within an organization and externally with the larger community. Even consortia, such as MELLODDY, facilitate federated learning, preserving confidentiality while pooling data across companies to improve ML models (for some; Blackburn-Owkin, 2022). Similarly, improvement of hardware and a growth in CROs providing infrastructure and workflow solutions make computation more accessible than ever. Nevertheless, the training of revolutionary transformer-based large language models like GPT-3 can cost tens of thousands or even millions in US$, requiring both big data and “big compute” (Sharir et al., 2020). Companies and institutions with the means to generate data at a scale beyond what is available to the public and those who can afford the compute requirements to train and deploy models at scale, will have a significant advantage over others. Though many large models are hosted openly for use, this also raises a reproducibility challenge as only those with access to big data and big compute will be able to validate them, compromising a key tenet of the scientific method.

The future of drug discovery will bring disruptive technologies and data that are unimaginable to us today. While it is possible that future AI will deliver a new generation of medicines, the belief that it will do so independently belies the complexity of the drug discovery process and undervalues the many scientific teams that contribute required inputs. Employing frameworks of cultural and data preparedness can ready us to tap into the data we have today with sustainable, thoughtful application of AI, and improve our probability of success in impacting human health through therapeutics.
